# Single-cell sequencing reveals cellular differences and potential mechanisms in congenital pulmonary airway malformation

**DOI:** 10.3389/fmed.2025.1548177

**Published:** 2025-06-03

**Authors:** Jinxi Huang, Qiang Chen, Songming Hong, Junjie Hong, Hua Cao

**Affiliations:** ^1^Department of Cardiothoracic Surgery, College of Clinical Medicine for Obstetrics & Gynecology and Pediatrics, Fujian Medical University, Fuzhou, China; ^2^Fujian Children’s Hospital (Fujian Branch of Shanghai Children’s Medical Center), Fuzhou, China

**Keywords:** CPAM, single-cell sequencing, KEGG, inflammation response, DEGs

## Abstract

**Objectives:**

Congenital pulmonary airway malformation (CPAM) is a common fetal lung developmental abnormality whose pathological process is not fully understood.

**Method:**

Single-cell sequencing technology is a novel high-throughput method that can reveal differences between different cell types and their role in disease.

**Results:**

By analyzing single-cell sequencing data from CPAM lesion tissue and normal tissue, we found an increase in erythrocytes, plasma cells and mast cells in CPAM samples. Additionally, genes such as CCL5, NKG7, GZMB, and SCGB1A1 were highly expressed in CPAM lesion tissues. The differentially expressed genes in CPAM samples are mainly related to functions such as inflammatory response, tissue remodeling, and immune response. Moreover, analysis of the signaling pathways involved in these differentially expressed genes revealed that pathways such as lysosome, phagosome, adherens junction, focal adhesion, and protein processing in the endoplasmic reticulum may be associated with the pathological process of CPAM.

**Conclusion:**

This study provides an in-depth analysis of the cellular differences between CPAM tissues and normal tissues using single-cell sequencing technology, revealing key cell types and functions involved in the development of CPAM. These findings provide important clues for a better understanding of the pathological process of CPAM and serve as a basis for identifying potential therapeutic targets.

## 1 Introduction

Congenital pulmonary airway malformation (CPAM) is a congenital anomaly that affects lung development (1). It is associated with abnormal cell proliferation and airway differentiation during fetal lung tissue development, and the prevalence of CPAM is approximately 0.81 per 10,000 fetuses (2, 3). Although CPAM has a relatively low incidence rate, it has a significant impact on the health and quality of life of affected children. CPAM is characterized by the presence of numerous cysts and abnormal airways in the lung tissue, which can be classified into four types (type I to type IV) based on the size and distribution of the cysts, with type I being the most common (4, 5). Patients with significant symptoms may require surgical removal of the abnormal tissue through thoracic surgery to relieve respiratory symptoms, infection, and organ compression, with lobectomy remaining the standard of care for surgical resection in most patients (6). The exact mechanisms underlying the development of CPAM are not fully understood, but studies have suggested that gene mutations during lung development may be involved (7, 8). Activation of the Wnt/β-catenin signaling pathway may play a critical role in CPAM development by regulating lung cell proliferation and differentiation, while the fibroblast growth factor (FGF) family signaling pathway also has important regulatory functions in embryonic development and organ formation (9). Abnormal expression of FGF10 may disrupt lung development and contribute to the occurrence of CPAM. In addition to genetic mutations, environmental and genetic factors may also play a role in the pathogenesis of CPAM. Due to its rarity, there is still a limited understanding of the relevant biomarkers and target genes associated with CPAM.

Single-cell transcriptome sequencing is an advanced biological technique that enables the high-throughput acquisition of gene expression profiles from individual cells. Compared to traditional bulk sequencing techniques, single-cell transcriptome sequencing provides more detailed and accurate information, allowing us to gain insight into gene expression differences within different cell subpopulations. Single-cell sequencing technology has been widely used for disease monitoring and mechanistic exploration, such as capturing the cellular composition of different tumors to reveal the heterogeneity of the tumor microenvironment (10). Although Tan et al. (11) identified SPOCK2 as a novel biomarker gene for CPAM, CPAM is a rare disease, and current research on CPAM cells and their characteristics at the single cell level is far from sufficient to explore its pathological mechanism. Therefore, to better understand the development and mechanisms of CPAM, we used single-cell transcriptome sequencing technology to analyze CPAM and normal healthy lung tissue samples. We performed quality control, integration and normalization of single-cell transcriptome data. Using dimensionality reduction and clustering analysis, we identified different cell subtypes and further analyzed differentially expressed genes among these subpopulations. The results revealed differences in cell types and relative proportions between CPAM and normal samples, and identified key molecules and target genes in CPAM, such as CCL5, NKG7, GZMB, and SCGB1A1. These results provide important clues and a bass for exploring the pathological mechanisms, etiology, and treatment methods of CPAM.

## 2 Materials and methods

### 2.1 Sample collection

**Figure 1:**
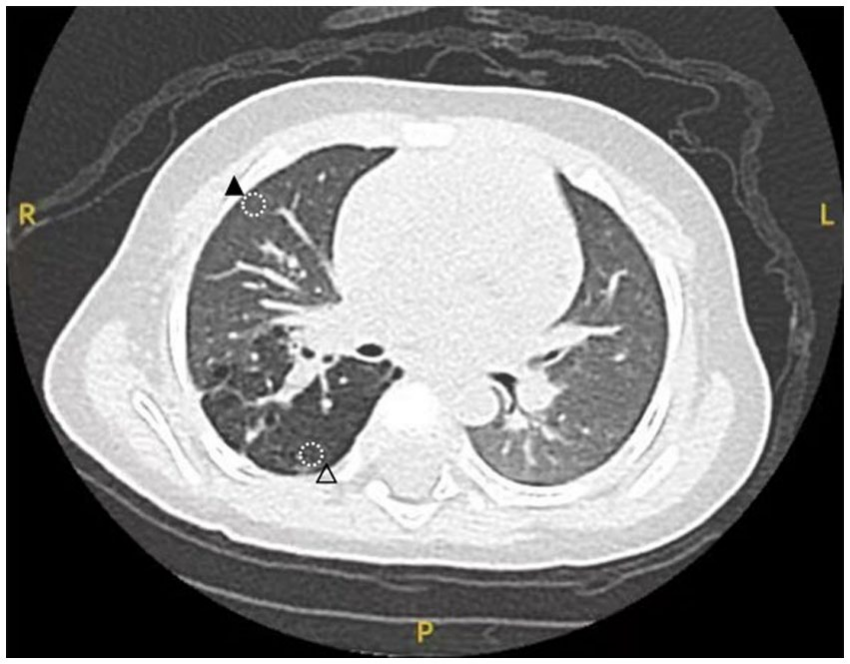
Preoperative CT image. ▲: Normal tissue sampling area. △: Lesion tissue sampling area.

The medical record included in this study pertains to a 5-month-old male infant who was diagnosed with a right thoracic lesion during the fetal period (at 23 weeks of gestation), suspected to be CPAM. The infant had no obvious respiratory distress after birth, and there was no history of recurrent respiratory infections from birth to the preoperative period. The general condition before surgery was good, with a weight of 7.5 kilograms. Computed tomography (CT) scans revealed a relatively large lesion in the right upper lung, classified as type II according to the Stocker classification. A right upper lobectomy was elected. Two sampling sites (lesion and normal tissue) were planned based on the CT images (Figure 1). Intraoperatively, the corresponding positions were marked with an electrocoagulation hook. The steps of lobectomy were as follows: first, the right upper pulmonary vein was exposed and clamped, then it was cut; each artery of the right upper lung was isolated and clamped before it was cut; the right upper bronchus was exposed and clamped, normal ventilation of the right middle and lower lung was confirmed, and finally the bronchus was cut. Three samples were collected from normal regions of the lung tissue, normal tissue was defined as regions distal to the lesion, confirmed via preoperative CT and intraoperative visual inspection. After removal of the lesion, two tissue samples measuring 0.5 cm × 0.5 cm × 0.5 cm were collected from the marked sampling sites for pathologic examination. After confirming the accuracy of the sampling area, an additional one tissue sample was taken from the same location and sent for single-cell sequencing analysis. All samples were obtained from the same patient. This study was conducted in strict accordance with the tenets of the Declaration of Helsinki and was approved by the Ethics Committee of Fujian Children’s Hospital, Approval Number: 2022ETKLR08053.

### 2.2 Single-cell sequencing workflow

Lesion and normal tissue samples (*n* = 3) are dissociated into a single-cell suspension using sCelLiVE^™^ tissue dissociation solution (nuclear isolation). Cells are diluted to an appropriate concentration, typically 2.5–3.5 × 10^5^ cells/mL. The cell suspension is loaded into the SCOPE-chip^™^ microfluidic chip, where individual cells are isolated according to the Poisson distribution principle. The cells fall under gravity into specialized microwells, ensuring that each well contains only one cell. Next, millions of magnetic beads bearing unique cell barcodes are added to the microwells, ensuring that each well contains only one bead. After cell lysis, the unique cell barcode and molecular marker (UMI) magnetic beads capture mRNA by binding to the poly(A) tails. This allows labeling of both cells (nuclei) and mRNA. The beads captured on the chip are collected, and the mRNA captured by the beads is reverse transcribed into cDNA and amplified. The cDNA is then fragmented, ligated with adapters, and prepared into sequencing libraries suitable for the Illumina sequencing platform. Single-cell sequencing well be performed by Nanjing Tongyuan Medical Laboratory (Nanjing, China).

### 2.3 Analysis of single-cell transcriptome libraries

For each individual sample, the following analyses are performed in sequence: data quality control analysis (removal of doublets and low quality cells based on certain thresholds), cell gene expression QC (display of nFeatureRNA, nCountRNA, mt.percent, etc.), cell clustering, cell type visualization by dimensional reduction (tSNE, tUMO), automated cell type annotation, cell clustering, cell type visualization by dimensionality reduction (tSNE/UMAP), automated cell type annotation, differentially expressed gene analysis between cell types, differentially expressed gene visualization, cell–cell interaction analysis (inferring interactions between different cell types), and enrichment analysis.

### 2.4 Statistical analysis

All statistical analyses were conducted using R software (version 4.0.5). DEGs between cell populations were identified using Wilcoxon rank-sum tests, with *p*-values adjusted via Bonferroni correction. Adjusted *p*-values <0.05 were considered statistically significant. The differences in cell proportions between CPAM and normal tissues were assessed using *χ*^2^ tests. Enrichment analyses for KEGG and GO pathways were considered statistically significant if the adjusted *p*-value was <0.05.

## 3 Results

### 3.1 Effect of CPAM lesion on lung tissue

**Figure 2:**
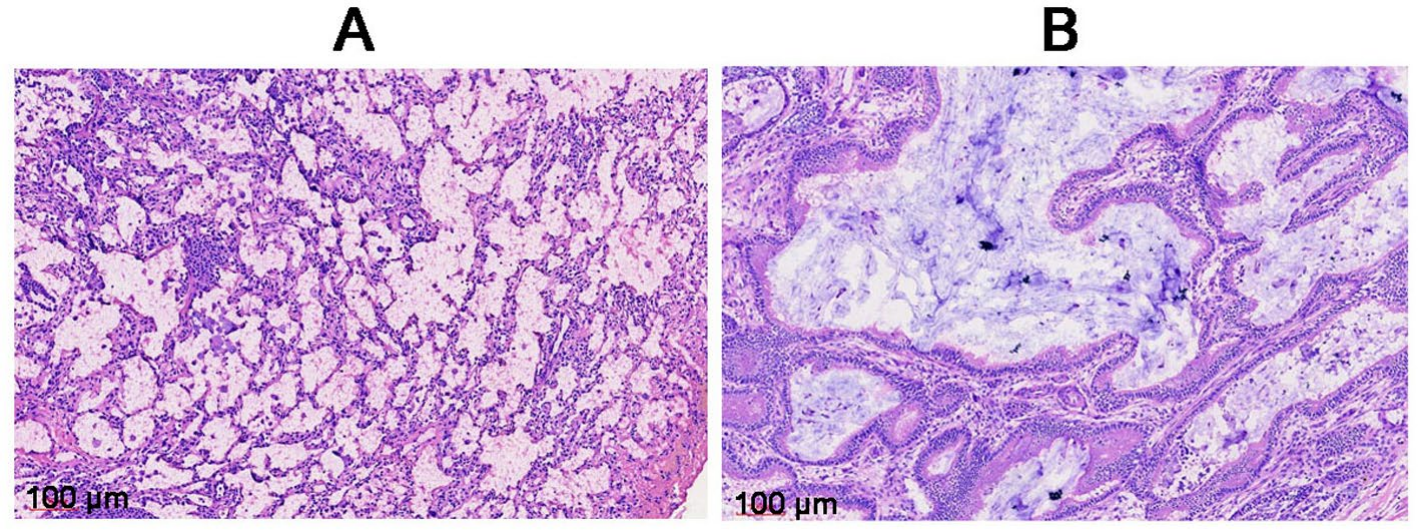
**(A)** Histopathological section of the normal tissue sampling area. **(B)** Histopathological section of the lesion tissue sampling area. Inflammatory infiltrates, including lymphocytes and macrophages, are observed in the fibrotic areas. Lymphocytes, typically small, round cells with a darkly stained nucleus and minimal cytoplasm, are concentrated in areas of chronic inflammation. Macrophages, in contrast, appear as larger cells with an irregular shape, abundant cytoplasm, and a less intensely stained nucleus.

Tissues affected by CPAM show prominent cavities containing minimal amounts of proteinaceous material. Healthy lung tissue consists of alveoli with alveolar walls populated by alveolar epithelial cells and capillaries. In CPAM-affected tissue, both cystic and non-cystic regions demonstrate cellular proliferation, with cell density exceeding that of healthy lung tissue and manifesting fibrosis, leading to a disorganized cellular arrangement. Furthermore, these CPAM-affected tissues also show signs of chronic inflammation, characterized by infiltration of lymphocytes and macrophages (Figure 2).

### 3.2 Distribution and characteristics of cells in CPAM tissue samples

**Figure 3:**
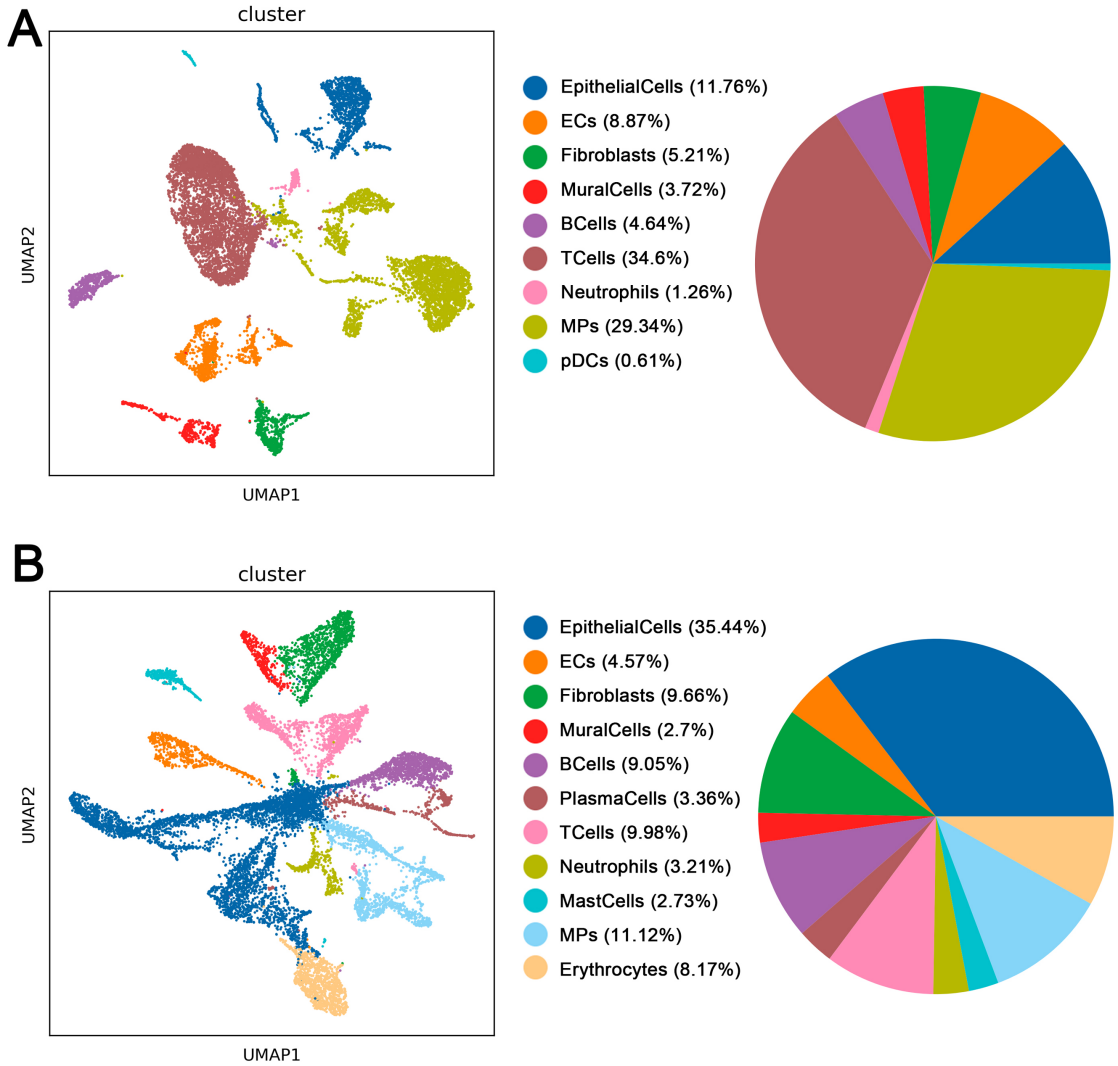
**(A)** Cell types in normal tissue of CPAM patients. **(B)** Cell types in lesion tissue of CPAM patients.

Through single-cell sequencing of CPAM and normal healthy samples, followed by quality control, integration and normalization processes, we performed dimensionality reduction clustering on the selected cells. We found significant differences in the distribution and location of cell clusters between CPAM and normal samples (Figure 3). In the healthy samples, we identified nine major cell types. Among them, T cells accounted for the largest proportion with 34.6%. This was followed by microphage cells (MPs) at 29.34%, epithelial cells at approximately 11.76%, endothelial cells (ECs) at 8.87%, fibroblasts at 5.21%, B cells at 4.54%, mural cells at 3.72%, neutrophils at 1.26%, and plasmacytoid dendritic cells (pDCs) at 0.61%. We identified 11 major cell types in CPAM samples. Among them, epithelial cells had the highest proportion with 35.44%. This was followed by MPS with 11.12%, T cells with 9.98%, fibroblasts with 9.66%, B cells with 9.05%, erythrocytes with 8.17%, endothelial cells with 4.57%, plasma cells with 3.36%, neutrophils with 3.21%, mast cells with 2.73% and mural cells with 2.7%. It is worth noting that CPAM samples showed an increase in the presence of erythrocytes, plasma cells and mast cells, and a lack of pDCs compared to normal healthy samples. There are clear differences in the composition of cell clusters between CPAM and normal samples. The observed changes in cell types and relative proportions in CPAM samples, particularly the increase in erythrocytes, plasma cells, and mast cells, may be related to the inflammatory response and tissue remodeling processes in CPAM pathology.

### 3.3 Enrichment analysis of differential genes in different cell subpopulations

**Figure 4:**
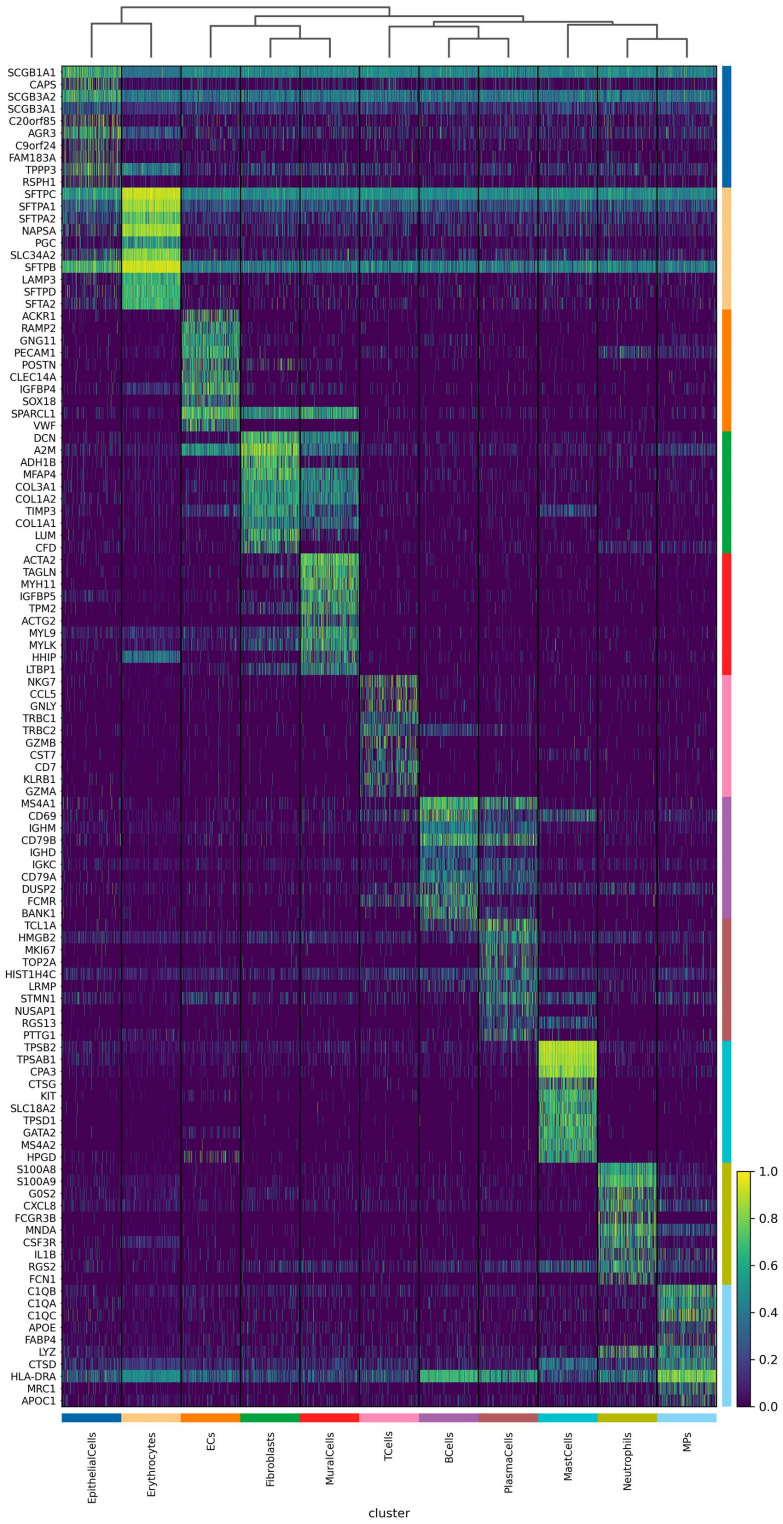
Differential genes contained in various cell types in lesion tissue of CPAM patients.

**Figure 5:**
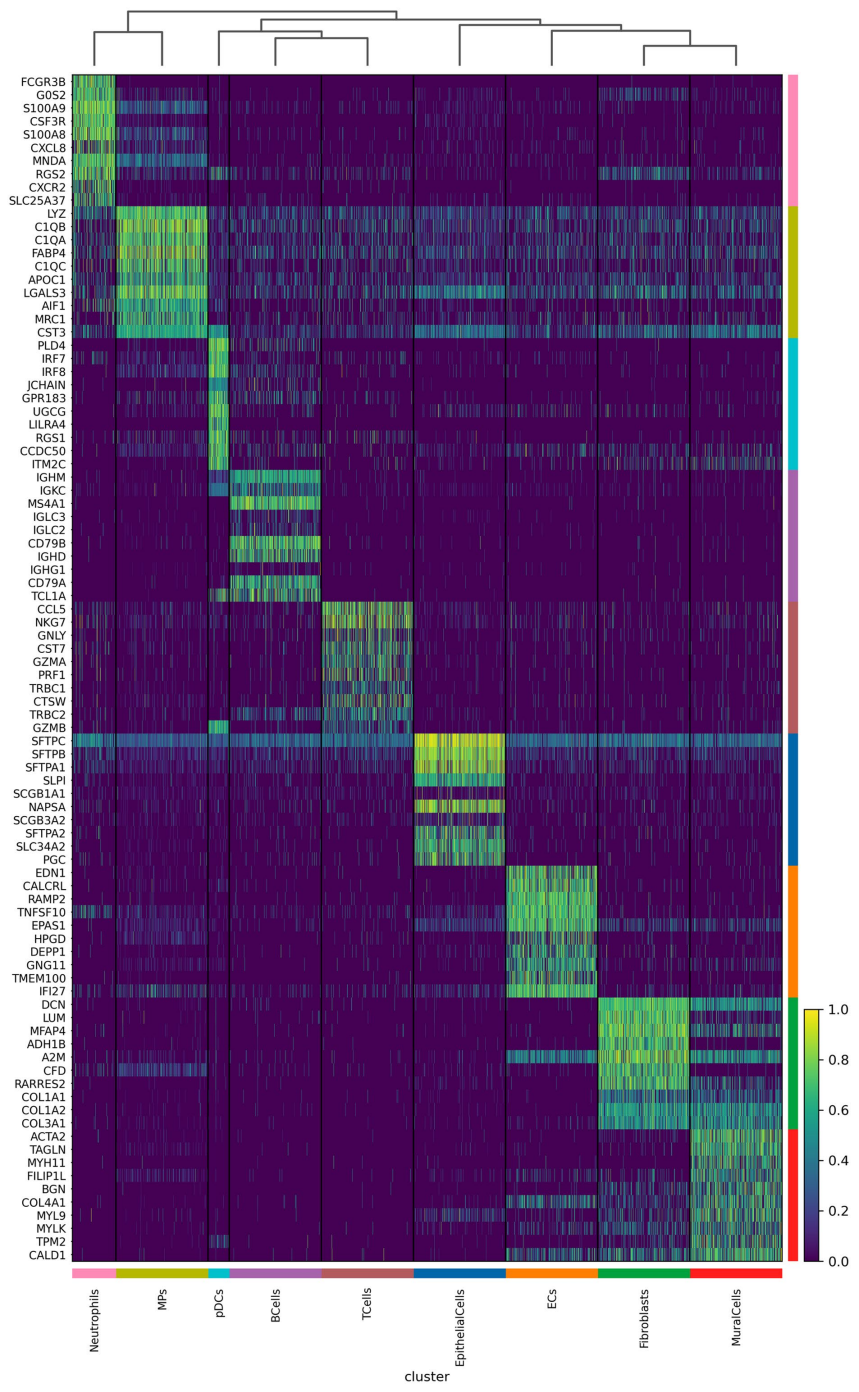
Differential genes contained in various cell types in normal tissue of CPAM patients.

We further analyzed the important differentially expressed genes in different cell subpopulations of CPAM (Figures 4, 5). The results showed that in CPAM samples, the top 10 differentially expressed genes in T cells were mainly NKG7, CCL5, GNLY, TRBC1, TRBC2, GZMB, CST7, CD7, KLRB1, and GZMA. In contrast, in healthy samples, the top 10 differentially expressed genes in T cells were mainly CCL5, NKG7, GNLY, CST7, GZMA, PRF1, TRBC1, CTSW, TRBC2, and GZMB. In CPAM samples, the top 10 differentially expressed genes in epithelial cells were mainly SCGB1A1, CAPS, SCGB3A2, SCGB3A1, C20ORF85, AGR3, C9ORF24, FAM183A, TPPP3, and RSPH1. On the other hand, in healthy samples, the top 10 differentially expressed genes in epithelial cells were mainly SFTPC, SFTPB, SFTPA1, SLPI, SCGB1A1, NAPSA, SCGB3A2, SFTPA2, SLC34A2, and PGC. In CPAM samples, the top 10 differentially expressed genes in MPS were mainly C1QB, C1QA, C1QC, APOE, FABP4, LYZ, CTSD, HLA-DRA, MRC1, and APOC1. Conversely, in healthy samples, the top 10 differentially expressed genes in MPS were mainly LYZ, C1QB, C1QA, FABP4, C1QC, APOC1, LGALS3, AIF1, MRC1, and CST3. In addition, we paid special attention to the differentially expressed genes in CPAM-specific cell subsets, including erythrocytes, plasma cells, and mast cells. In erythrocytes, the differentially expressed genes mainly included SFTPC, SFTPA1, SFTPA2, NAPSA, PGC, SLC34A2, SFTPB, LAMP3, SFTPD, and SFTA2. In plasma cells, the differentially expressed genes were mainly TCL1A, HMGB2, MK167, TOP2A, HIST1H4C, LRMP, STMN1, NUSAP1, RGS13, and PTTG1. In mast cells, the differentially expressed genes mainly included TPSB1, TPSAB1, CPA3, CTSG, KIT, SLC18A2, TPSD1, GATA2, MS4A2, and HPGD. The expression levels of differentially expressed genes in various cell subgroups showed significant changes between CPAM samples and healthy samples. These differentially expressed genes are involved in immune responses, airway epithelial cell functions, clearance of particulate cells, and regulation of inflammation, among others.

### 3.4 KEGG and GO enrichment analysis screened important signaling pathways

**Figure 6:**
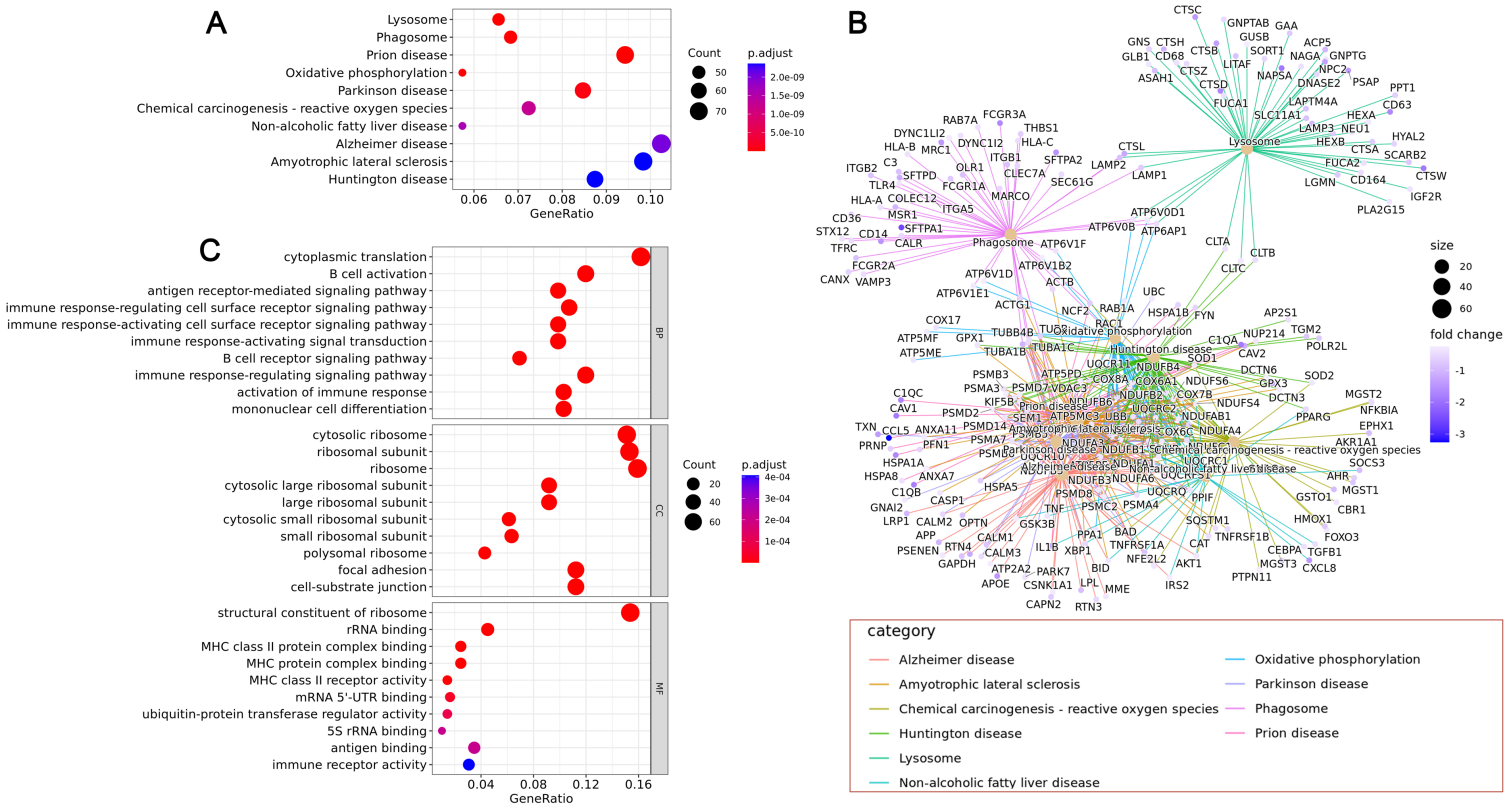
Differential gene expression and function in normal tissue of CPAM patients. **(A)** KEGG enrichment analysis of differential gene signaling pathways in normal tissues of CPAM patients. **(B)** Interaction network among differential genes. **(C)** GO analysis of the enrichment of differential genes in BP, CC, and MF.

**Figure 7:**
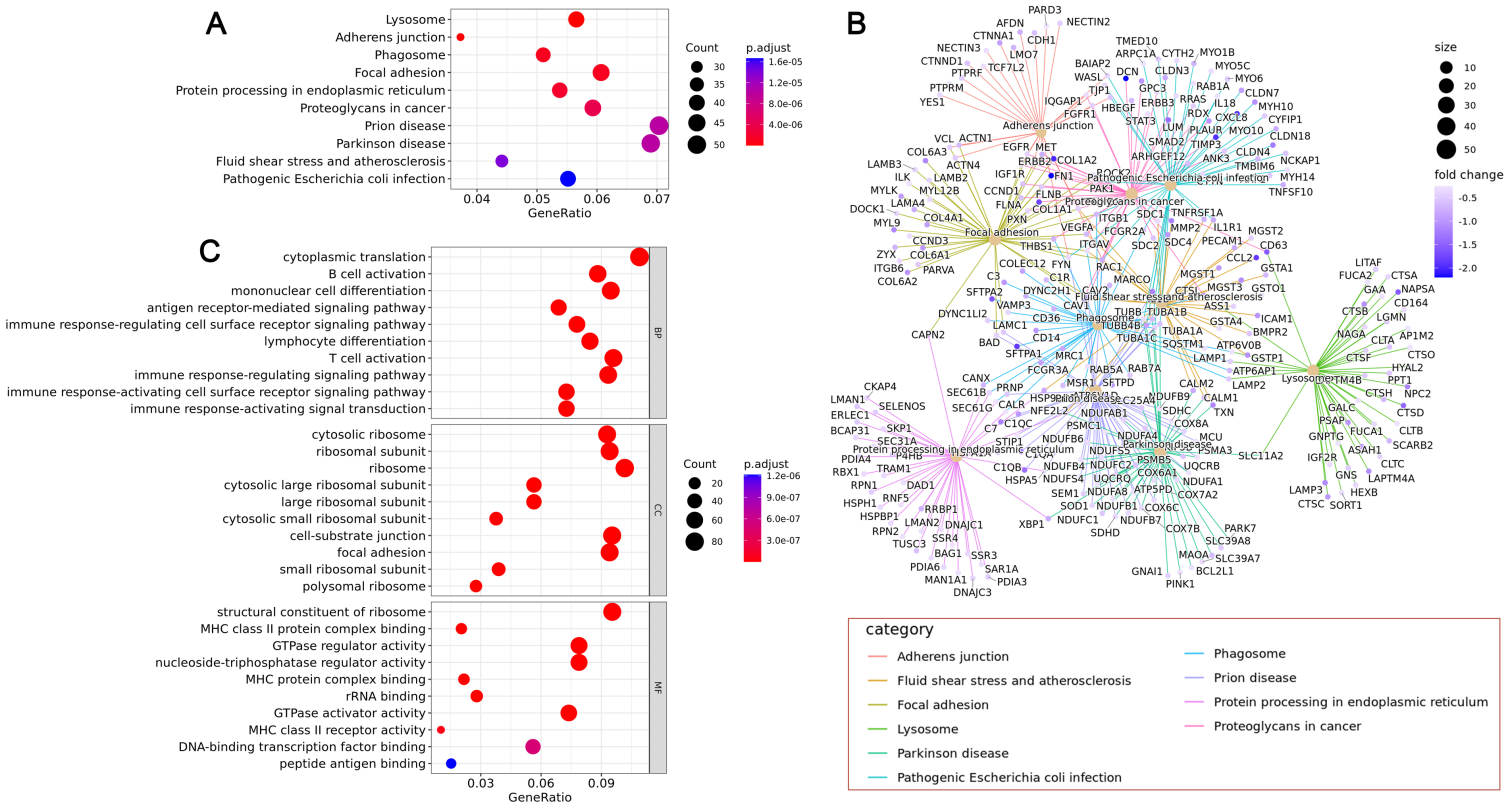
Differential gene expression and function in lesional tissue of CPAM patients. **(A)** KEGG enrichment analysis of differential gene signaling pathways in lesional tissues of CPAM patients. **(B)** Interaction network among differential genes. **(C)** GO analysis of the enrichment of differential genes in BP, CC, and MF.

By performing enrichment analysis on the upregulated genes in different cell types, we can identify significant correlations between these cell type-specific highly expressed genes and specific biological functions or pathways. Using clusterProfiler software for KEGG pathway enrichment analysis of gene sets, the results show that in healthy samples, the most significant pathways are lysosome, phagosome, prion disease, oxidative phosphorylation, and Parkinson’s disease. In the CPAM tissue region, the most significant pathways include lysosome, adherens junction, phagosome, focal adhesion, and protein processing in the endoplasmic reticulum (Figures 6A, 7A). Through further analysis of these enrichment results, we have uncovered the potential interactions among differential genes within the CPAM-associated aberrant biological functions and pathways, which constitute a significant influence on the pathogenesis and progression of CPAM (Figures 6B, 7B). GO analysis indicates that in the realm of biological processes (BP), processes related to immune responses, such as T cell activation, B cell activation, and antigen receptor-mediated signaling pathways, are significantly enriched in CPAM tissues. This suggests the possibility of active immune cells and inflammatory processes in CPAM tissues; in terms of cellular components (CC), changes in intracellular structures, such as those involving ribosomal subunits and structures connected to the extracellular matrix (e.g., cell-matrix junctions), may reflect an increase in protein synthesis activities or alterations in cell adhesion properties within CPAM tissues; molecular function (MF) reveals the activated molecular activities in CPAM tissues, like binding activities of MHC class molecules and GTPase-related functions, implying the vigor of immune presentation and signaling transduction processes (Figures 6C, 7C).

## 4 Discussion

Based on the single-cell sequencing results of CPAM samples and normal healthy samples, we found significant differences in cell populations between CPAM and normal samples. The increased presence of erythrocytes, plasma cells, and mast cells in CPAM samples may be related to inflammatory responses and tissue remodeling in which CPAM types play important roles, such as erythrocytes carrying oxygen (12), plasma cells producing antibodies, and mast cells mediating inflammatory responses (13). In T cells, there is partial overlap in differentially expressed genes between CPAM and healthy samples. Genes such as CCL5, NKG7, and GNLY show significant differential expression in both sample groups, and these genes are associated with immune response and cytotoxic activity, with higher expression levels in CPAM samples, possibly reflecting an increase in the severity of inflammatory responses (14–16). In epithelial cells, there is also partial overlap in differentially expressed genes between CPAM and healthy samples. The differentially expressed genes in CPAM samples are mainly related to genes associated with airway epithelial cells, such as SCGB1A1, SCGB3A2, AGR3, and increased expression levels of these genes may be associated with proliferation and differentiation of lung adenoma cells (17, 18). The involvement of Th17 cells in CPAM pathology may be aligned with the cell type’s known role in chronic inflammatory immune-mediated diseases. Th17 cells are crucial in maintaining mucosal barriers and contribute significantly to the pathogenesis of various chronic inflammatory conditions, including autoimmune diseases (19). In our study, the differential expression of genes associated with T cell activation in CPAM samples could reflect similar inflammatory mechanisms mediated by Th17 cells, contributing to the persistent inflammation observed in CPAM. This observation is consistent with findings from chronic inflammatory diseases, where Th17 responses play a key role in promoting inflammation and tissue remodeling, which could parallel the fibrotic changes and immune dysregulation noted in CPAM. Zhang et al. (20) performed a comprehensive single-cell transcriptome analysis that revealed the mechanism of abnormal proliferation in epithelial cells in congenital cystic adenomatoid malformation. Their results identified key gene expression changes in epithelial cells, consistent with our findings regarding the involvement of SCGB1A1 and SCGB3A2 in epithelial cell dysregulation in type II CPAM. In healthy samples, genes closely related to surfactant synthesis and alveolar function, such as SFTPC, SFTPB, and SFTPA1, have higher expression levels. MPS is a cell type involved in phagocytosis, antigen presentation and regulation of inflammatory responses. Macrophages were annotated based on the high expression of C1QA, C1QB, APOE, and LYZ. In CPAM samples, differentially expressed genes in MPS are mainly involved in immune regulation and anti-inflammatory processes. In healthy samples, differentially expressed genes mainly include C1QB, C1QA, and APOC1 (21, 22), which are involved in phagocytic cell clearance function and regulation of inflammatory responses. Functional enrichment analyses of differentially expressed genes in macrophages further confirmed their roles in phagocytosis, antigen presentation, and immune regulation, as highlighted by the expression of genes such as C1QB, C1QC, and APOE.

For CPAM samples with erythrocytes, plasma cells and mast cells, we observed some differentially expressed genes. In erythrocytes, the expression levels of some surfactant-related genes (such as SFTPC, SFTPA1, SFTPA2) are increased, which may be related to neovascularization and early lung developmental abnormalities (23, 24). In plasma cells, differentially expressed genes are associated with cell proliferation and immune response, such as TCL1A, HMGB2, etc. (25, 26). In mast cells, differentially expressed genes are mainly associated with mast cell activation and regulation, such as KIT (27), MS4A2, etc. By analyzing the differentially expressed genes, CCL5, NKG7, GZMB, and SCGB1A1 can be considered as potential targets for CPAM. For example, the study by Tan et al. (11), which integrated bulk and single-cell RNA sequencing, identified SPOCK2 as a critical gene involved in CPAM type II. Their results align with our discovery of inflammation-related genes such as CCL5 and NKG7, which also indicate immune dysregulation in CPAM. By analyzing the signaling pathways involved in the differentially expressed genes, we found abnormal biological functions and pathway changes that may occur during the development of CPAM. For example, the enrichment of lysosome and phagosome pathways may indicate abnormal intracellular metabolism and degradation related to the pathological process of CPAM. Li et al. (28) conducted an integrative analysis combining bulk and single-cell RNA sequencing and identified critical signaling pathways, including Wnt/β-catenin and FGF, as being central to CPAM pathogenesis. These findings align with the pathway enrichments observed in our study, particularly in relation to tissue remodeling and immune response pathways such as adherens junction and focal adhesion. Enrichment of adherens junction and focal adhesion pathways may indicate changes in cell adhesion, tissue structure, and cell-matrix interactions associated with CPAM development. Enrichment of protein processing in the endoplasmic reticulum pathway may reflect the importance of endoplasmic reticulum dysfunction in the onset and development of CPAM.

The study has several limitations, including a sample size of a single patient, which restricts generalizability due to potential patient-specific variations and the rarity of the condition. Additionally, the CPAM stage (type II) and sampling site selection may introduce bias, as pathological heterogeneity across CPAM subtypes or sampling regions could influence the results. Future studies with larger cohorts and diverse CPAM subtypes are needed to validate these findings. Furthermore, we did not conduct validation of single-cell sequencing *in vitro* or in animal models, including investigations into potential key genes, signaling pathways, and immune cell mechanisms. These aspects will need to be addressed in future research.

## 5 Conclusion

Using single-cell sequencing technology, we can comprehensively and systematically analyze the differences in cell populations between CPAM tissues and normal tissues, and reveal key cell types and functions involved in CPAM development. This will provide important clues for a deeper understanding of the pathological process and potential therapeutic targets of CPAM. Although the analysis of differentially expressed genes can provide candidate targets and key pathways, functional verification is still required to confirm their exact roles in CPAM development. Further experimental studies can include *in vitro* and *in vivo* models to validate whether these genes and pathways have critical effects on the occurrence and development of CPAM, thereby providing a comprehensive understanding of the CPAM development mechanism.

## Data Availability

The original contributions presented in the study are publicly available. The datasets used in this study can be found in the NCBI SRA repositories (Reference No. PRJNA1263638).
